# Impact of Hydrogen-Enriched Natural Gas on the Accuracy of Odorant Measurements

**DOI:** 10.3390/s25144394

**Published:** 2025-07-14

**Authors:** Giorgio Ficco, Viviana Cigolotti, Gino Cortellessa, Giulia Monteleone, Marco Dell’Isola

**Affiliations:** 1 Department of Civil and Mechanical Engineering, University of Cassino and Southern Lazio, 03043 Cassino, Italy; g.cortellessa@unicas.it (G.C.);; 2 Department of Energy Technologies and Renewable Sources, Agenzia Nazionale per le Nuove Tecnologie, l’Energia e lo Sviluppo Sostenibile (ENEA), Research Center Casaccia, 00123 Rome, Italy

**Keywords:** hydrogen blending, odorization, methane, odorant measurement, odorant concentration

## Abstract

Blending hydrogen with natural gas is emerging as a pivotal strategy in the transition to low-carbon energy systems. However, the exploitation of the natural gas infrastructure to distribute natural gas and hydrogen blends (and 100% hydrogen in the long-term) introduces several technical, economic, and safety issues. These latter are paramount, especially in urban distribution networks that supply residential buildings and dwellings, since the quality and safety of the living environment can also be significantly affected. In this scenario, the reliability of odorant concentration measurements according to the best practices currently in use for natural gas becomes crucial. This study is aimed at assessing the accuracy of odorant measurements at different concentration levels (i.e., low, medium, and high) in 100% methane, methane–hydrogen blend and 100% hydrogen. The obtained results show the tendency to overestimate the odorant concentration up to 2.3% in methane–hydrogen blends at medium and high concentrations of THT as well as the underestimation of −3.4% in 100% hydrogen at low concentration of TBM. These results are consistent with those of natural gas from the city distribution network with hydrogen content of 5% and 20%.

## 1. Introduction

Blending hydrogen into natural gas networks represents a promising practice to reduce carbon emissions while leveraging existing infrastructure without significant modifications to pipelines or appliances. However, this transition necessitates addressing technical challenges, such as material compatibility and safety standards [1,2]. In fact, by utilizing current pipelines and distribution systems, hydrogen blending provides a feasible path to lower emissions in sectors reliant on natural gas, such as power generation, heavy industry, heating, and transport [3]. However, achieving optimal integration requires addressing technical challenges, including adjustments to gas composition, compatibility with pipeline materials, and safety standards. Nazanin et al. [4] claim relevant progress in the development of injection strategies, mixing solutions, sensors, and materials have been observed in recent years. However, although numerous numerical studies exist, experimental research on mixing and injection systems remains limited, particularly on the open research issues relating to sensors capable of operating in high-pressure transmission pipelines, and material solutions such as coatings that can inhibit embrittlement of pipelines. Aminul et al. [5] investigated the effect of hydrogen blends in a range of materials spanning vintage steels, cast irons, aluminum, plastics, elastomers, and more modern materials and highlighted the necessity of updating technical codes and standards to ensure safe operation with hydrogen blends.

In this context, the reliability of odorant concentration measurement systems emerges as an open research theme. Odorization is crucial for safety, since natural gas, as well as hydrogen, is colorless and odorless. In the EU, natural gas odorization is always mandatory in distribution networks, unlike transmission, where it is mandatory only in some member states (i.e., Spain, Greece, Ireland, Romania, Sweden). The odorant level is normally set in terms of minimum and maximum concentration (mg/Sm^3^), since too low concentrations can cause safety problems while too high concentrations can cause operational problems [6]. It is also necessary to avoid odorant overdose to contain costs and environmental impact. The costs of overdose also include those associated with the greater impact on the infrastructure (i.e., the corrosion of pipelines and regulation and measurement devices) as well as those associated with potential false alarms. K. Pradheep et al. [7] investigated the stability and homogeneity of hydrogen blends in low-pressure pipelines, concluding that hydrogen did not significantly separate or react under typical flow conditions, and the odorants currently used in distribution networks remain detectable. S. Navroop et al. [8] documented that odorants would separate from the CH_4_ and H_2_, following many hours of stagnation. They also conclude that the odorant (TBM) would still be detectable at levels comfortably above the human detectability threshold.

Technical and regulatory standards generally mandate that gas must be odorized in such a way that its presence in the atmosphere is easily detectable at all gas concentrations and not lower than one fifth of the lower flammability limit, ensuring that a person with a normal sense of smell can identify it. Consequently, the presence of natural gas at 1% in the air must be detected by smell [9,10,11]. As for example, in Italy technical standard UNI 7133-2 [12] defines the minimum concentrations of odorant (i.e., 32 mg/Sm^3^ for THT and 9.3 mg/Sm^3^ for TBM as major component of the mixture with IPM and NPM). Depending on the type and characteristics of the distribution network, the odorant concentration may not be homogeneous at all points, due to the network configuration and orography as well as the downstream consumption regimes. In [13], the results of a numerical simulation (based on the Navier–Stokes equations and the k-ε turbulence model) of the optimal odorant concentration in a City Gate Station (CGS) of a distribution network in Iran are presented. In particular, the effects of the pipeline length, the gas velocity as well as the temperature and pressure of the gas on the initial and final odorant concentration were investigated. The results show that the odorant concentration decreases with increasing pipeline length and gas velocity. Similarly, as the gas temperature increases or the pressure decreases, the gas velocity increases and, consequently, the odorant concentration decreases. The authors also propose a correlation to calculate the final odorant concentration as a function of the natural gas velocity, the initial odorant concentration at the beginning of the pipeline and the length of the pipeline.

In the view of injection of hydrogen into natural gas grids, the need to study the impact of hydrogen on odorization practices emerges. Aiming at ensuring the reliability of the distribution service, the Regulatory Authority has set specific gas quality standards in terms of Higher Calorific Value (HCV) components and trace components and physical properties [14]. It can be underlined that, in compliance with the MITE Decree of 3 June 2022 [15], the Network Code currently in force in Italy [16] provides a 2% limit for hydrogen in natural gas. On the technical side, there does not appear to be any significant issues of chemical compatibility between hydrogen and commonly used odorants. However, aiming at distributing natural gas and hydrogen blends, the Distribution System Operator (DSO) will likely need to review its odorant injection strategy and determine whether it is necessary to increase odorant injection rates to account for the dilution effect of hydrogen blending.

Recent studies suggest that sulfur compounds, such as THT, can effectively odorize natural gas and hydrogen mixtures without undesired chemical reactions with hydrogen under typical distribution network conditions. In this regard, it should be considered that the lower flammability limit for natural gas is approximately 4.4% vol and is substantially similar to that of hydrogen (approximately 4% vol) but varies with the gas composition. Thus, this does not appear to pose a significant issue for natural gas and hydrogen mixtures, in particular for the definition of minimum concentration limits to ensure leak detectability. In Italy, the established minimum odorant concentrations have been tested by ITALGAS Reti with methane and 20% hydrogen and by HERA with natural gas and 30% hydrogen. The results of these experiments (partly also conducted in the presence of the interferent DMS at a concentration of 60 mg/Sm^3^) allow the confirmation of the minimum odorant concentrations also in the case of hydrogen injection of up to 20% vol [12].

In Europe, several research projects are investigating the odorization of natural gas and hydrogen mixtures using traditional odorants without encountering significant problems [17,18]. In [19], it is affirmed that mixtures of hydrogen and natural gas require a slightly higher odorant concentration than that needed for natural gas alone. Typically, for mixtures containing 20% hydrogen an increase of about 4% is estimated (i.e., from 6 to 6.25 mg/Sm^3^). The odorization of the mixture can be achieved by odorizing the hydrogen directly at the hydrogen supply point or in the blending unit or indirectly by increasing the odorant injection rate. However, if hydrogen was to be odorized directly, an odorant concentration of 7.3 mg/Sm^3^ would be needed, since 100% hydrogen has a lower flammability limit than natural gas (4% vs. 4.4%). In [20], five odorants, both those for use in gas networks (including THT and a so-called “new blend”, which is a mixture of 78% TBM and 22% DMS) and those declared compatible for use with fuel cells, were tested to check their compatibility for use with 100% hydrogen blends. The results show that four odorants met the requirements for gas distribution networks in the UK and only one (i.e., 5-ethylidene-2-norbornene) did not fully pass the test. Huszal and Jaworski [21] evaluated the reliability of natural gas odorant measurement and control systems in the presence of compositional variations, for example, resulting from the injection of up to 15% hydrogen, confirming the absence of significant impacts on the quality of the measurement results obtained using two types of chromatographic analyzers. The same authors, in [22], presented and discussed the results of experimental research on the impact of hydrogen injection on the stability of the THT odorant, demonstrating the absence of interaction between hydrogen (up to 15% vol) and THT in the group 2E network gas. In [23], it is affirmed that the effectiveness of the odorants used in natural gas networks is substantially not influenced by hydrogen injection. Finally, in [24] the ability to detect gas leaks by untrained people has been investigated with odorized hydrogen against odorized methane and natural gas. The results show that the odorants currently used for natural gas will have similar effectiveness when used with hydrogen and that small hydrogen leaks are detectable in a comparable way to natural gas leaks in the same room volume. In summary, the literature suggests that blends with lower hydrogen concentrations (e.g., below 20%) typically maintain adequate odorization with conventional methods, though a higher hydrogen content can affect both the odor detectability and dispersion. As hydrogen content increases, so does the challenge of maintaining consistent odor perception, which may require new measurement standards.

This work is aimed at addressing a critical gap in the available scientific literature. In fact, while existing studies predominantly focus on the compatibility and usability of odorants in hydrogen-enriched gas mixtures, in this work for the first time the accuracy and reliability of measurement systems for odorant concentration are investigated, aiming at addressing the following open research questions:

- Are existing odorant measurement technologies and devices reliable with hydrogen-enriched gas mixtures?
- Does hydrogen blending could cause inaccurate readings due to altered gas matrix or interference with sensors?

To this aim, an experimental campaign has been carried out and the accuracy of odorant concentration measurements has been evaluated both in laboratory and in the field. Therefore, this study not only sheds light on the operational challenges of integrating hydrogen into existing gas infrastructure but also provides actionable insights for enhancing safety and reducing costs. It is worth underlining that the overestimation of odorant concentration can lower the ability to detect gas leaks, whereas the underestimation can lead to false alarms and cause increasing costs for ensuring minimum concentration threshold in the gas. The results of this research could be useful for setting hydrogen blending policies and for utility companies for revising procedures for odorant dosing and monitoring to meet future compliance. On the other hand, the limitation of this study is represented by the fact that, due to the difficulty of injecting hydrogen into a real city network, experimental measurements have been conducted with certified gas cylinders (for methane-hydrogen gas blends) and with natural gas from a city distribution network delivered to an industrial user (for natural gas–hydrogen blends), which represent a sort of rated operating condition. This reduces the degree of proximity to real operating conditions.

## 2. Methods

Sulfur compounds including tetrahydrothiophene (THT) and various mercaptans (e.g., ethyl mercaptan and tert-butyl mercaptan) were traditionally used to add a penetrating odor to the gas, making it easily detectable. Today, mixtures of multiple components are usually used to achieve the desirable characteristics. Sulfur-based odorants such as THT or TBM do not react with hydrogen and are believed to be effectively used in gas distribution networks. Table 1 summarizes the main characteristics of the most common odorants used in natural gas grids.

In Italy, technical standard UNI 7133-2 [12] establishes the basic criteria for the odorization of fuel gases and describes the methods for measuring the odorant concentrations aiming at guaranteeing the gas itself has a sufficient odor intensity. The systems for monitoring the odor intensity and the odorant concentration in fuel gases are also described. The latest revision of this standard extends the scope of application to mixtures of natural gas and hydrogen. Furthermore, periodic measurements of the odor intensity and/or the degree of odorization of fuel gases must be carried out at least twice a year, as required by UNI 9463-2 [25], in correspondence with the seasonal regimes of high and low flow rates for gases distributed via pipes. It is common practice to perform tests using gas chromatographic methods that evaluate the concentration of the odorant in the unit of volume of the fuel gas. The rhino-analytical method in [26] is also permitted. In this regard, it is worth noting that analytical methods such as GC focus on chemical composition whereas olfactory methods focus on the human sensory experience. In [27], a review of the most common sensory methodology used for quantitative and qualitative assessment of odor intensity and concentration has been carried out, highlighting that field studies are the least biased except for odor concentration, where the dispersion modeling approach also showed great potential. On the other hand, Byliński et al. [28] compared GC methods and field olfactometry in a vicinity of an oil refinery and a wastewater treatment plant noting higher values of odor concentration were obtained with the field olfactometry technique.

Aiming at measuring odorant concentrations in natural gas, chromatographic techniques are applicable (e.g., flame photometric, electrochemical, and thermal conductivity detectors) that normally guarantee a good separation of the components of analytical interest through the appropriate combination of gas chromatographic columns and selective or non-selective detectors. In [12], it is also permitted that non-gas chromatographic instrumental methods are applicable if their effectiveness in terms of validation process and uncertainty estimation is demonstrated. In [29], some requirements for the determination of sulfurous compounds in natural gas are also defined.

Both continuous and spot analytical measurements can be employed to enable ongoing monitoring of odorant concentration levels directly in the field, such as at regulating and measuring stations of the DSO. In this process, a sample from the gas flow is analyzed immediately by a measuring device installed within the network, available on an equipped vehicle, or transported to a laboratory for testing. Recent advances in portable gas chromatography (GC) systems, facilitated by integrating microfabricated components, have rendered these instruments ideal for rapid, in-field analyses of complex chemical mixtures, including natural gas in pipelines. Portable GC systems, often equipped with mass analyzers, are capable of detecting THT (at 5 ppm) or TBM (in the range of 1–3 ppm) directly within the pipeline, achieving performance levels comparable to laboratory instruments. However, the use of such instrumentation on the field poses several challenges, including the need for a continuous supply of carrier gas, maintenance of the columns (with periodic self-calibration), and the potential for variations in operating conditions to impact measurement accuracy. As a result, spectroscopy can offer a viable alternative method for these analyses.

In [30], a new approach for the detection of TBM and THT in odorized natural gas by UV-visible (UV-Vis) spectrophotometry is proposed, applicable in a wide concentration range (from about 10^−3^ to thousands of mg/Sm^3^). In fact, methane, which is the main component of natural gas, exhibits UV absorptions up to 140 nm, as well as propane and ethane (up to 150 nm), while sulfur compounds absorb UV light in the spectral region above 195 nm. In [31], it is demonstrated that the use of the infrared absorption spectroscopy method with radiation sources in the spectral range 6–15 μm allows for obtaining an odor detection sensitivity of about 5 ppm in gas mixtures of different compositions. Currently, gas chromatography remains the most accurate and reliable method for the detection of odorant in natural gas even in complex matrices and at low concentration, thanks to its high sensitivity (sub-ppm to ppb range) and selectivity. However, some drawbacks emerge for the on-field use of GC with an automatized sampler for continuous analyses, such as the need for a constant flow of carrier gas, the unavoidable release into the atmosphere of the quantities of gas analyzed. On the other hand, IR absorption could be useful for qualitative analysis, whereas UV Spectroscopy, despite the latest developments, still presents strong limitations (e.g., TBM and THT do not have strong UV-absorbing groups, low reliability without derivatization).

Recently, the accredited PT Provider UNICHIM conducted an interlaboratory test with 12 participating laboratories aimed at assessing the reliability of odorant measurement in natural gas [32]. To this aim, the composition and odorization of a synthetic mixture simulating a natural gas containing hydrogen, C_2_–C_6_ hydrocarbons, nitrogen, carbon dioxide, and odorant (TBM and THT) was measured. The 12 laboratories measured the odorant content (TBM and/or THT) using different detectors by applying methods consistent with [29]. Only in one single case with TBM a value outside the acceptability limits was found, demonstrating the good reproducibility of the methods and systems used for measuring the odorant concentration.

It is well known that mixing hydrogen with natural gas modifies the physical properties of the gas [33], such as density and vapor pressure, with possible effects on the effectiveness of the odorant and on the reliability of the odorant concentration measuring devices. Even minor changes in gas mixture composition can determine variations in chemical–physical properties such as density, dynamic viscosity, Joule-Thomson coefficient, heat capacity, thermal conductivity, volumetric energy density, and vapor-liquid equilibrium that are crucial for the safe management of network infrastructures. Considering the effects of hydrogen in the mixture, the choice of an appropriate odorant is crucial, since some odorants can react with hydrogen and/or alter the properties of the gas itself. For example, odorants with lower density and higher vapor pressure could be better suited to higher quantities of hydrogen. Indeed, the significant differences in the properties of hydrogen and methane (the main component of natural gas), as briefly summarized in Table 2, can lead to unexpected issues in the management and balancing of gas pipelines and for end-user applications when mixtures of hydrogen and natural gas are supplied.

It is noteworthy that as the hydrogen content in the mixture increases, the flame temperature increases and the flammability limit is extended. In particular, the increase in the flame temperature can determine an increase in nitrogen oxide emissions and for this reason the use of mixtures with increasing hydrogen concentrations requires greater measures for the reduction in emitted NOx. The extension of the flammability limits combined with a reduction in the activation energy [34] can also determine a greater instability of the mixture. Finally, an increase in hydrogen at the same volumetric flow rate determines a reduction in the overall energy content supplied. A further critical issue is that when hydrogen is mixed with natural gas, the density of the mixture is lower than that of the natural gas, with the effect that gas losses along the network can increase [35]. In this scenario, Gas Chromatographs are expected to reliably detect hydrogen contents in the gas stream but may require different columns/detectors for accurate quantification. Also, a recalibration for blended gases should be beneficial, since aging effects on columns and detectors may be enhanced by the hydrogen.

In the present research, a test campaign was specifically designed to experimentally investigate the accuracy of odorant concentration measurement when hydrogen-enriched natural gas is used. To this aim, reference measurement data (e.g., those obtained by certified gas cylinders) were compared with experimental data obtained by applying in the laboratory and in the field current techniques and available instruments for odorant concentration measurements. The test campaign consisted of two phases: (i) tests on certified cylinders with methane and hydrogen blends; (ii) tests on odorized natural gas delivered by a city network and hydrogen blends in a scaled network at an industrial site. It is worth noting that real network conditions such as pressure and temperature and pipeline type and aging have not been considered as influencing factors at this stage of the research. Future developments of this research will include field tests under real conditions. Also, a digital model of a portion of an urban gas grid will be realized aimed at simulating real conditions in some critical points (i.e., flow, temperature, pressure, odorant concentration, hydrogen content) in line with recent developments of the system analysis method in the gas industry [36,37].

Experimental tests were carried out by the Gas Chromatographic Analysis Laboratory for Odorization of Pietro Fiorentini Spa, San Vito al Tagliamento, Italy. The laboratory is accredited with the number 1293L by ACCREDIA for the execution of tests to measure the intensity and TBM/THT odorant concentration in the range 5–100 mg/Sm^3^ in gas mixtures for domestic use. To this aim, technical standards [12] and [29] are referred and a Gas Chromatograph (GC) Agilent 490 Micro GC (Agilent Technologies Inc. Santa Clara, CA, USA) for odorant measurements was used. The GC for odorant measurements relies on two separate injectors: (i) Channel 1: CP-Sil 19CB Heated Injector (TCD) for THT, (ii) Channel 2: CP-Sil 13CB Heated Injector (TCD) for TBM. Before the experimental measurements, a two-step calibration of the GC has been carried out by using pressurized cylinders, the first with natural gas from three different compositions and the second with mixtures of natural gas and hydrogen at 5%, 10%, and 15%, and 100% H_2_, respectively. Calibration of the GC has been performed with Helium as a carrier gas (in the flow-rate range within 4.0 mL/min) and at a column temperature *t_c_* = 90 °C and pressure *P_c_* = 200 kPa. Separate calibration curves have been used for the investigated CH_4_-H_2_ 20% mixture and for 100% H_2_. This procedure ensures that the GC operates within the prescribed accuracy limits of class 0.5. Finally, each experimental measurement was made up of a series of at least 10 repeated measurements every 15 min.

### 2.1. Tests on Methane/Hydrogen Blends in Certified Cylinders

The first phase of the measuring campaign was carried out using certified cylinders at three odorant THT/TBM concentration values, i.e., low–medium–high (about 5, 30 and 100 mg/Sm^3^) each in three gas mixtures (i.e., 100% CH_4_, 100% H_2_ and a mixture 20% H_2_ and 80% CH_4_). Measurements with 100% CH_4_ were used for the in-field validation of the measuring process in actual test conditions. The low–medium–high concentrations chosen can be considered typical, respectively, of a terminal branch of the network with low consumption downstream (e.g., in summer), of a meshed network and of a section immediately downstream of the odorization plant with high consumption (e.g., in winter).

**Figure 1:**
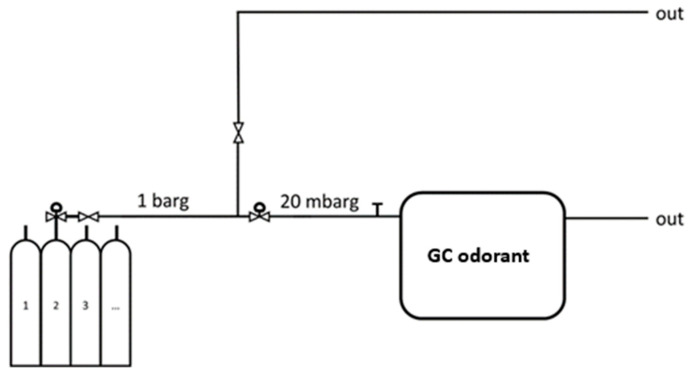
Schematic diagram of experimental layout for tests on certified cylinders.

In Table 3, the details of the tests carried out are reported, including the indication of the certified reference value with the related measurement uncertainty. The investigated gas mixtures and odorant concentration were certified by the SIAD laboratory (SIAD Group, Osio Sopra, Italy) accredited by ACCREDIA as reference materials producer (RMP). Finally, Figure 1 shows a diagram and the test layout for the measurements on certified cylinders.

### 2.2. Tests on Natural Gas from the Gas Grid with Hydrogen Injection

**Figure 2:**
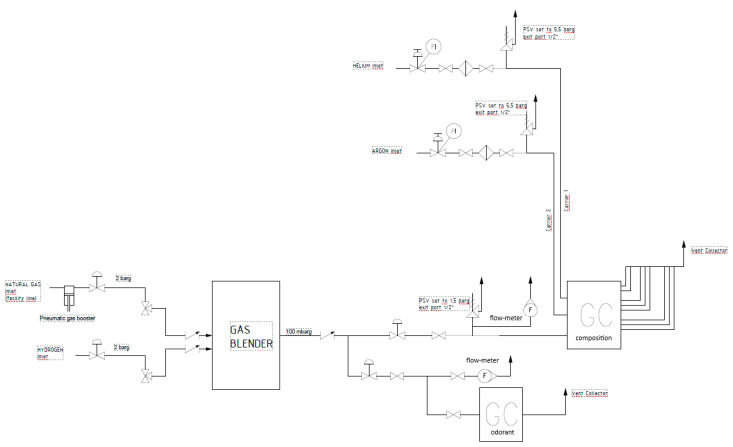
Experimental layout for tests in natural gas and hydrogen blends.

The second phase of the measuring campaign was carried out at the Hydrogen Innovation Lab of Pietro Fiorentini Spa, Arcugnano, Italy [38], using the natural gas odorized with THT delivered by the city distribution network. At the inlet of the Lab the natural gas from the network is pressurized up to 5 bar and subsequently injected at 2 bar into the gas blender. In addition to the odorized natural gas, the gas blender is also supplied with pure hydrogen from a cylinder at 2 bar. In such configuration, the odorized natural gas and pure hydrogen are mixed in the desired proportions (i.e., 5% and 20% of hydrogen) into the gas blender. The resulting mixture is immediately measured downstream of the blending unit and then vented. The test layout is illustrated in Figure 2, including the auxiliary instrumentation employed during the experiments.

### 2.3. Calculations

The relative error ($E_{\%}$) was calculated using Equation (1), where $I_{GC}$ is the GC indication and $C_{ref}$ is the reference concentration value of the certified cylinder.

(1) $$E_{\%}=\frac{I_{GC}-C_{ref}}{C_{ref}}\times 100$$

For tests on natural gas from the gas grid, however, since a certified reference value was not available, an expected reference value was estimated. For this purpose, having measured a variable THT concentration in the city network, the mean measured values were used to derive a linearization function in order to obtain a $C_{GN,i}$ value interpolated at the exact time of measurement on the NG- H_2_ mix. Consequently, the expected reference value $C_{ref}$ of the THT concentration is estimated starting from the H_2_% vol hydrogen content measured in the mixture, using Equation (2).

(2) $$C_{ref}=C_{GN,i}\;(1-\frac{{H2}_{\%vol}}{100})$$

Finally, the normalized error $E_{n}$ between reference and measured values for tests on certified cylinders has been calculated through Equation (3), in which $C_{odor}$ and $C_{ref}$ are the measured and the reference odorant concentrations and $U_{odor}$ and $U_{ref}$ their respective expanded uncertainties. According to [39], the measurement performance can be considered as satisfactory when $E_{n}\leq$ 1.

(3) $$E_{n}=\frac{\left|C_{odor}-C_{ref}\right|}{\sqrt{U_{odor}^{2}+U_{ref}^{2}}}$$

### 2.4. Uncertainty Estimation

The expanded uncertainty ($U_{odor}$) of the odorant concentration measurement was estimated considering the following contributions:

- The standard uncertainty of the reference value ($u_{ref}$) obtained from the certificate of the cylinder used;
- The standard uncertainty of the GC, $u_{GC}$ = accuracy/$\sqrt{3}$, considering that the GC used conforms class 0.5;
- The standard uncertainty of the GC calibration, $u_{cal}$ = 0.15 mg/Sm^3^ as per calibration certificate;
- The repeatability uncertainty ($u_{rep}$) equal to the standard deviation of the repeated measurements performed (>10 measurements).

By applying the uncertainty propagation law and considering a coverage factor k = 2 corresponding to an approximately 95% confidence interval, the expanded uncertainty of the odorant concentration is given by the following Equation (4).

(4) $$U_{odor}=k\sqrt{u_{ref}^{2}+u_{GC}^{2}+u_{cal}^{2}+u_{rep}^{2}}$$

## 3. Results and Discussion

### 3.1. Methane/Hydrogen Blends in Certified Cylinders

**Figure 3:**
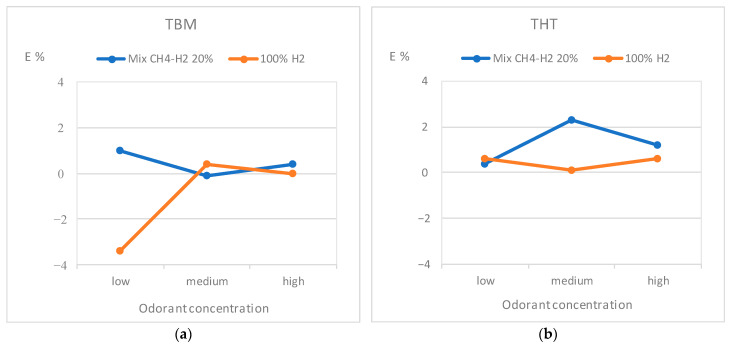
Mean error as a function of odorant concentration and gas mixture: (**a**) TBM, (**b**) THT.

Table 4 and Table 5 show the results of the experimental tests carried out on certified cylinders, respectively, for the measurement of TBM and THT, while in Figure 3 the same results are reported in a graphic form. $C_{odor}$ is the average value of at least ten readings.

From the analysis of the results, it is worth noting that initial tests with 100% CH_4_ showed measured errors always within ±0.1% both for THT and TBM, thus confirming the consistency of the measuring process with the accuracy class 0.5 for odorant. For tests with the CH_4_-H_2_ blend at 20% vol of hydrogen, the tendency to overestimate the concentration was found for THT at medium ($E_{\%}\;$= 2.3%) and high concentrations ($E_{\%}$ = 1.2%), whereas no particular issues have been found for TBM at all the investigated concentrations and for THT at low concentration. Moreover, for tests with 100% H_2_ all positive errors were found, generally within 0.6%. Therefore, these errors are almost consistent with the GC class 0.5, except for TBM at low concentration for which an error $E_{\%}$ = −3.4% was found. T-test for assessing the statistical significance of the differences between gas mixtures has been carried out, demonstrating no significant differences arising between the odorant (i.e., TBM/THT) nor between the mixtures (i.e., CH_4_-H_2_ blend at 20% vol of H_2_ and 100% H_2_), since calculated *p*-values were always above 0.05. On the other hand, the significant statistical difference is highlighted for different odorant concentrations (i.e., low, medium, high concentration), showing the different behavior of the measuring device when the concentration varies. Furthermore, no critical issues emerged from the analysis of outliers using the IQR (Interquartile Range) method. Finally, it can be pointed out that in all the tests carried out, low values of $E_{n}$ (i.e., within 0.34 and 0.38 for TBM and THT, respectively) were found, demonstrating the reliability of the odorant concentration measurement even with hydrogen blends and 100% hydrogen. The above reported results seem consistent with those in [21,22] for THT and CH_4_-H_2_ 20% mixture and in [7] for sulfur compounds.

In relation to the slight underestimation of −3.4% measured for TBM at 100%, this could be ascribed to the interaction between odorant and hydrogen, as well as material properties and limitations in measurement devices. While no direct degradation of the sensors was observed during our experimental campaign, such underestimation suggests potential interaction mechanisms, possibly reversible and surface-related, rather than chemical degradation. Traditional fade mechanisms in natural gas (i.e., adsorption on pipe walls, oxidation by rust, absorption into condensates and masking effects) also apply to hydrogen odorant streams; however, no direct data currently compare fade rates in 100% H_2_ vs. NG systems. Hydrogen can permeate materials more easily than methane, potentially affecting how odorants behave. The recent research project Hy4Heat [17] did not report significantly higher adsorption for selected odorants in 100% H_2_ for relatively short-term (e.g., 8 h). On the other hand, based on experimental laboratory tests, Huszal and Jaworski [21] claim hydrogen does not chemically react with THT, but they raised concerns that hydrogen could influence odorant performance via processes like adsorption onto pipeline surfaces, since odorant concentration depends heavily on surface interactions. Hydrogen, being a small and highly diffusive molecule, may exacerbate this phenomenon by facilitating stronger or more frequent interactions between odorants and surfaces. Finally, at the instrument level, hydrogen’s low-viscosity and high-diffusivity can mean that odorants may travel unevenly in the injected plug and partition unpredictably at the inlet, which can change peak shape and affect measurement accuracy. While there is no significant degradation of GC components, the measurement properties can shift due to changes in carrier gas matrix. Therefore, we recommend recalibration of GC systems when switching to hydrogen-enriched mixtures. However, experimental evidence relating to odorant fading and measurement accuracy in 100% H_2_ stream is still lacking. Moreover, GC may not be accurately calibrated for 100% hydrogen, as hydrogen can affect the baseline, sensitivity, or retention time of odorants during measurement. Finally, odorant injection could not be always optimal, and incomplete mixing may lead to uneven odorant distribution, and sampling may not reflect actual concentrations. In this sense, optimizing odorant injection systems for hydrogen’s properties, ensuring complete and rapid mixing of odorants with hydrogen, could be beneficial. Moreover, the use of hydrogen-compatible materials like stainless steel or fluoropolymers can help reduce measurement errors, in addition to performing calibration and validation of gas chromatographs and detectors using standards in hydrogen matrices, not methane or air.

**Figure 4:**
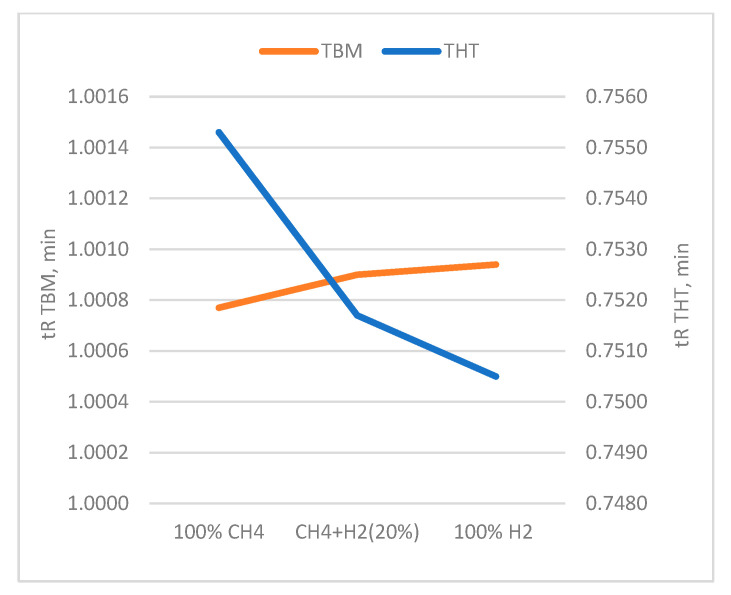
Trend of the retention time $t_{R}$ as a function of the gas mixture.

Particularly noteworthy is the analysis of the retention time $t_{R}$. In chromatography, the retention time is the time taken between the injection of the sample and the recording of the peak maximum; it depends on the nature of the substance, the column, and the operating conditions. The corrected retention time $t_{m}^{\prime }$ = $t_{R}$ − $t_{m}$ is used, obtained by subtracting the dead time $t_{m}$; i.e., the time taken by the eluent alone to pass through the column and corresponding to the first peak present on the chromatogram. For the purposes of this study, retention times were manually corrected. Table 6 shows the values of $t_{R}$ measured during the tests on the certified cylinders, while in Figure 4, the same values are reported graphically.

However, the presence of hydrogen at high concentrations (e.g., up to 20%) in the analyzed sample mixture should not significantly interact with the stationary phase in most capillary columns. However, it can slightly alter retention behavior of light gases by displacing them or changing partition dynamics in packed columns. This could also be ascribed to the lower viscosity of hydrogen.

From the analysis of the data, it can be pointed out that the retention time is substantially unchanged for the measurements with TBM (i.e., variation within 0.02%), whereas for the measurements with THT the retention time decreases (i.e., variation within 0.64%) as the hydrogen content increases. Consequently, a calibration before using the GC in NG-H_2_ mixture is suggested.

### 3.2. Natural Gas from the Gas Grid and Hydrogen Blends

As a first step, the composition and THT odorant concentration of the test gas from the city distribution grid has been measured and, subsequently, those of the NG-H_2_ blends with 5% and 20% hydrogen. The test cycle ends with an additional measurement of the composition and THT odorant concentration of the gas from the urban grid to check possible drifts. Each measurement has been repeated at least 10 times to achieve repeatability estimation. In Table 7, a summary of the composition and properties of the test gases is reported, whereas, in Table 8 the obtained results are presented.

**Figure 5:**
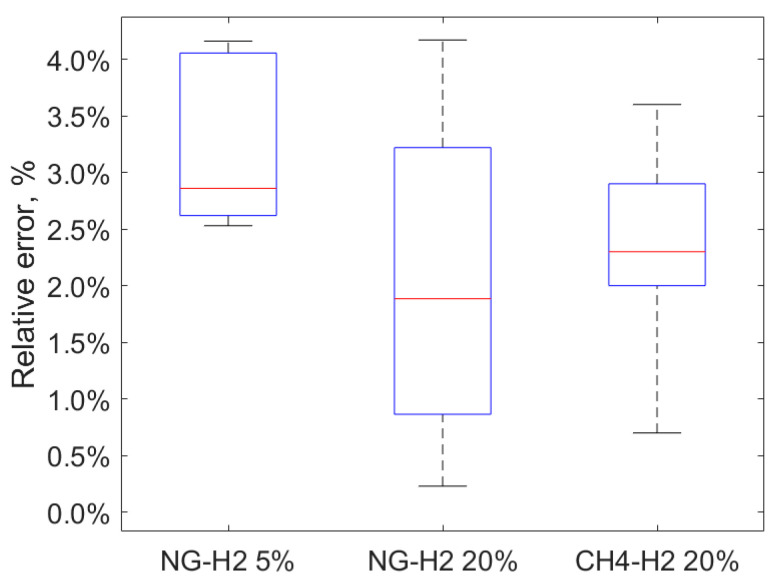
Box plot of the measured relative errors.

From the above reported results, a tendency to overestimate the odorant concentration was found, in both medium ($E_{\%}$ = 3.2%) and high concentrations of H_2_ ($E_{\%}$ = 2.0%). The results obtained are consistent with those for the CH_4_-H_2_ 20% vol mix measured with certified cylinders (see Figure 5). Therefore, the reliability of the methodology was demonstrated. However, the overestimation of the odorant concentration observed could pose risks, such as failing to adequately detect gas leaks or incurring unnecessary costs associated with maintaining odorant levels above the required minimum. These findings underscore the importance of accurate calibration and monitoring of odorant injection strategies in hydrogen-enriched natural gas systems.

## 4. Conclusions

The reliability of the measurement of odorant concentration has been investigated aiming at assessing the capability of current gas chromatographs with natural gas–hydrogen blends.

To this aim, tests in certified cylinders of methane, CH_4_-H_2_ blend, and pure hydrogen each at three different concentrations (i.e., low-medium-high) of TBM and THT odorants commonly used in natural gas grids have been conducted. The experimental results demonstrate the good reliability of odorant concentration measurement even with hydrogen blends and 100% hydrogen. However, measurements at 100% hydrogen in respect to CH_4_- H_2_ blend with 20% vol of hydrogen seem more accurate. In fact,

- the overestimation of 2.3% and 1.2% for THT, respectively, at medium and high concentrations occurs with CH_4_- H_2_ blend at a 20% vol of hydrogen, whereas no issues have been found for TBM (at all investigated concentrations) and THT at low concentration;
- errors generally within 0.6% were found for 100% hydrogen; however, such errors were all positive except for TBM at low concentration (i.e., $E_{\%}$ = −3.4%).

The tendency to overestimate the odorant concentration was also found for tests with natural gas from the city distribution grid, both at medium concentrations of H_2_ = 5% vol ($E_{\%}$ = 3.2%) and high concentrations of H_2_ = 20% vol ($E_{\%}$ = 2.0%). It is worth underlining that the overestimation (underestimation) of the odorant concentration can lead to the risk of not detecting gas leaks and unnecessary costs (spread of unjustified alarms and unnecessary costs for ensuring the minimum concentration level of odorant).

Finally, the retention time of the gas chromatograph remains substantially unchanged for the use with TBM, whereas for the measurements with THT a decrease as the percentage of hydrogen increases has been observed. Therefore, a specific calibration before using the GC in NG-H_2_ mixture should be beneficial.

The next stage of this research involves designing a test campaign using a large portion of a city network with the injection of hydrogen produced on-site at different percentages.

## Figures and Tables

**Table 1:** Main technical properties of odorants [6].

Odorant	Mol. Weight	THT + EA	THT + TBM	THT	TBM + IPM + NPM
THT (C_4_H_8_S)	88.2	12%	70%	100%	
EA (C_5_H_8_O_2_)	100.1	88%			
TBM (C_4_H_10_S)	90.2		30%		76%
IPM (C_3_H_8_S)	76.2				16%
NPM (C_3_H_8_S)	76.2				8%
Density at 273 K (kg m^−3^)	950		1016	825	
Density at 288 K (kg m^−3^)	910	893	1003	810	
Vapor pressure at 273 K (mbar)	11		5.8	82	
Vapor pressure at 288 K (mbar)	27	84	13	170	
% S	4.4	36.1	36.4	37.1	

**Table 2:** Properties of methane and hydrogen.

Property	Unit	Methane, CH_4_	Hydrogen, H_2_
Wobbe Index	MJ/Sm^3^	50.60	44.44
Lower Heat Value	MJ/Sm^3^	39.13	12.74
Density	Kg/Sm^3^	0.717	0.0899
Lower flammability limit	% vol	4.4	4.0
Higher flammability limit	% vol	17	75
Laminar flame velocity	cm/s	30–40	200–300
Adiabatic flame temperature	°C	1962.78	2204.44

**Table 3:** Investigated gas mixtures and cylinders.

Certified Cylinder	Odorization Level	Test Report	Reference Values	
			THT, mg/Sm^3^	TBM, mg/Sm^3^
100% Methane	Odor_low	C067023	5.04 ± 0.25	5.00 ± 0.49
	Odor_medium (THT)	C000824	31.8 ± 1.61	-
	Odor_medium (TBM)	C067123	-	30.2 ± 3.02
	Odor_high	C067223	100.48 ± 3.03	100.13 ± 4.97
80% CH_4_ + 20% H_2_	Odor_low	25143–SN013206	5.16 ± 0.29	5.12 ± 0.29
	Odor_medium	26459–SN014508	31.09 ± 1.27	31.7 ± 1.30
	Odor_high	26457–SN014507	108.0 ± 3.25	102.43 ± 3.13
100% Hydrogen	Odor_low	26460–SN013215	4.88 ± 0.27	4.95 ± 0.28
	Odor_medium	26459–SN014508	30.3 ± 1.20	30.0 ± 1.20
	Odor_high	26457–SN014507	101.0 ± 3.10	99.8 ± 3.10

**Table 4:** Test results on TBM certified cylinders (mg/Sm^3^).

Odor	100% CH_4_			Mix CH_4_-H_2_ 20%			100% H_2_		
	Low	Medium	High	Low	Medium	High	Low	Medium	High
$C_{odor}$	5.00	30.19	100.06	5.17	31.66	102.86	4.78	30.13	99.76
$U_{odor}$	0.58	3.04	5.02	0.43	1.36	3.24	0.42	1.26	3.18
$C_{ref}$	5.00	30.20	100.13	5.12	31.7	102.43	4.95	30	99.8
$U_{ref}$	0.49	3.02	4.97	0.29	1.30	3.13	0.28	1.20	3.10
E	0.00	−0.01	−0.07	0.05	−0.04	0.43	−0.17	0.13	−0.04
E%	0.0%	0.0%	−0.1%	1.0%	−0.1%	0.4%	−3.4%	0.4%	0.0%
$E_{n}$	0.00	0.06	0.04	0.10	0.02	0.10	0.34	0.07	0.01

**Table 5:** Test results on THT certified cylinders (mg/Sm^3^).

Odor	100% CH_4_			Mix CH_4_-H_2_ 20%			100% H_2_		
	Low	Medium	High	Low	Medium	High	Low	Medium	High
$C_{odor}$	5.04	31.84	100.50	5.18	31.82	109.31	4.91	30.33	101.61
$U_{odor}$	0.39	1.66	3.10	0.43	1.41	3.85	0.41	1.26	3.31
$C_{ref}$	5.04	31.8	100.48	5.16	31.09	108	4.88	30.3	101
$U_{ref}$	0.25	1.61	3.03	0.29	1.27	3.25	0.27	1.20	3.10
E	0.00	0.04	0.02	0.02	0.73	1.31	0.03	0.03	0.61
E%	0.1%	0.1%	0.0%	0.4%	2.3%	1.2%	0.6%	0.1%	0.6%
$E_{n}$	0.01	0.02	0.00	0.04	0.38	0.26	0.06	0.02	0.13

**Table 6:** Retention time $t_{R}$ (min) measured as a function of the mixture and odorant.

Retention Time $t_{R}$ (Min)	THT	TBM
$t_{R}$ for 100% CH_4_	0.7553	1.00077
$t_{R}$ for mix CH_4_ and 20%H_2_	0.7517	1.00090
$t_{R}$ for 100% H_2_	0.7505	1.00094

**Table 7:** Test gases composition and properties.

Description	Unit	Hgas (Gas Grid)	Hgas +5% H_2_	Hgas +20% H_2_
Methane	%	92.3934	86.9870	72.9875
Carbondioxide	%	0.8465	0.7978	0.6717
Ethane	%	5.3029	4.8591	4.1791
Propane	%	1.0442	0.9593	0.8259
i-Butane	%	0.1425	0.1347	0.1126
n-Butane	%	0.1730	0.1592	0.1360
i-Pentane	%	0.0398	0.0362	0.0311
n-Pentane	%	0.0307	0.0275	0.0240
C6+	%	0.0271	0.0258	0.0221
Hydrogen	%	0.0000	6.0134	21.0100
Nitrogen	%	0.0000	0.0000	0.0000
Total summed quantity	%	100	100	100
Higher heating value	kWh/Sm^3^	11.7011	11.1910	9.9755
Lower heating value	kWh/Sm^3^	10.5685	10.0950	8.9673
Wobbe index	kWh/Sm^3^	15.0336	14.7909	14.2138
Relative density	--	0.6058	0.5725	0.4926
Density	kg/Sm^3^	0.7833	0.7402	0.6369
Molar mass	kg/kmol	17.5073	16.5503	14.2522
Compressibility	--	0.9971	0.9975	0.9984
Odorant concentration (THT)	mg/Sm^3^	44.97	42.58	35.85

**Table 8:** Results of tests with natural gas from the grid and hydrogen blends.

	Hydrogen Blend	
	NG-H_2_ 5%	NG-H_2_ 20%
*C_mis_* (mg/Sm^3^)	42.58	35.85
*C_ref_* (mg/Sm^3^)	41.24	35.12
*E* (mg/Sm^3^)	1.34	0.72
*E%* mean	3.24%	2.04%
*E%* median	2.86%	1.88%
*E%* max	4.16%	4.17%
*E%* min	2.53%	0.23%

## Data Availability

Data will be made available on request.

## Abbreviations

The following abbreviations and symbols are used in this manuscript:

MA	methyl acrylate
EA	ethyl acrylate
DSO	Distribution System Operator
THT	tetrahydrothiophene
TBM	tert-butilmercaptano
CGS	City Gate Station
HCV	Higher Calorific Value
GC	gas chromatography
PT	proficiency test
NG-H_2_	natural gas and hydrogen mixture
E	error, mg/Sm^3^
$E_{\%}$	relative error, %
$E_{n}$	normalized error
$I_{GC}$	GC indication, mg/Sm^3^
$C_{ref}$	reference concentration value of the certified cylinder, mg/Sm^3^
$u_{ref}$	uncertainty of the reference value, mg/Sm^3^
$u_{GC}$	uncertainty of the GC, %
$u_{tar}$	uncertainty of the GC calibration, mg/Sm^3^
$u_{rep}$	repeatability uncertainty, mg/Sm^3^
$C_{odor}$	measured odorant concentration, mg/Sm^3^
$C_{ref}$	reference odorant concentration, mg/Sm^3^
$U_{odor}$	expanded uncertainty of the measured odorant concentration, mg/Sm^3^
$U_{ref}$	expanded uncertainty of the reference odorant concentration, mg/Sm^3^
$t_{R}$	retention time, min
